# Triptolide in the treatment of systemic lupus erythematosus - regulatory effects on miR-146a in B cell TLR7 signaling pathway in mice

**DOI:** 10.3389/fphar.2022.952775

**Published:** 2022-09-23

**Authors:** Yi Zhang, FengQi Zhang, YiNi Gao, MeiJiao Wang, Yan Gao, HaiChang Li, Jing Sun, ChengPing Wen, ZhiJun Xie

**Affiliations:** ^1^ School of Basic Medical Science, Zhejiang Chinese Medical University, Hangzhou, China; ^2^ The Second School of Clinical Medicine, Zhejiang Chinese Medical University, Hangzhou, China

**Keywords:** triptolide, systemic lupus erythematosus, B cell, miR-146a, toll-like receptor 7

## Abstract

**Objective:** To clarify the mechanism of triptolide (TP) in alleviating the conditions underlying SLE.

**Methods:** Eight-week-old MRL/lpr mice were randomly divided into a model group (*n* = 5), low-dose TP (TP-L) group (*n* = 5), and high-dose TP (TP-H) group (*n* = 5). Mice in these groups were gavaged with normal saline, low-dose TP solution, and high-dose TP solution for 8 weeks, respectively. The expression levels of anti-dsDNA, IgG, IgM, IgA, C3, C4, and CREA, BUN, ALT, AST, ALB, and ALP indexes in the serum of mice were detected. The proportion of CD19^+^CD138^+^B220^−^ cells in the spleen and the pathological changes of kidney tissue in the mice were also evaluated. The possible signaling pathways and microRNA (miRNA) targets of TP in the treatment of SLE were analyzed using network pharmacology. The expressions of TLR7 mRNA and miR-146a in Raji cells (a B lymphocyte line) were detected using qPCR before and after intervention with a miR-146a inhibitor. The protein expression levels of TLR7, MyD88, p-IRAK1, and p-NF-κBp65 were detected using western blot analysis.

**Results:** TP could significantly decrease the levels of ds-DNA and IgG, alleviate pathological injury in renal tissue, and upregulate miR-146a expression in the B cells of MRL/lpr mice without obvious liver and kidney toxicity. Network pharmacology analysis showed that TP could mainly regulate the Toll-like receptor signaling pathway, and NF-κB signaling pathway, among others. miRNA target prediction suggested that TP could regulate miRNAs such as miR-146a. *In vitro* cell experiments further confirmed that TP could significantly upregulate miR-146a expression and downregulate the expression of TLR7 mRNA and protein levels TLR7, MyD88, p-IRAK1, and p-NF-κBp65. After intervention with a miR-146a inhibitor, TP had no obvious inhibitory effects on TLR7, MyD88, p-IRAK1, and p-NF-κBp65 expression.

**Conclusion:** TP may exert therapeutic effects on SLE by regulating miR-146a expression, inhibiting the TLR7/NF-κB signaling pathway, and affecting B cell activation.

## Introduction

Systemic lupus erythematosus (SLE) is characterized by the production of autoantibodies. It is a chronic autoimmune disease that seriously affects the quality of life of patients. B lymphocytes proliferate after being stimulated by antigens and produce specific antibodies. The current treatment paradigm in SLE revolves around effectively regulating B cell activation and reducing the production of autoantibodies (Liossis and Melissaropoulos, 2014). It is worth noting that Toll-like receptor 7 (TLR7) is crucial for the development of this B cell population (Rubtsov et al., 2011). TLR7 is an endosomal Toll-like receptor recognizing single-stranded RNA (ssRNA) (Diebold et al., 2004). Overexpression of the RNA-recognizing TLR7 has been linked to SLE in humans and mice (Weindel et al., 2015). On the one hand, the excessive activation of TLR7 is thought to be involved in the pathogenesis of SLE (Souyris et al., 2018). It has been shown that the overexpression of TLR7 induces systemic autoimmunity in a lupus-prone mouse strain (Walsh et al., 2012). On the other hand, the deletion of TLR7 reduces the development of lupus in strains that spontaneously develop the disease (Christensen et al., 2006). Moreover, over the past few years, significant advances have been made in understanding how TLR7 functions in B cells promote autoimmune disease (Satterthwaite, 2021). Thus, TLR7 signaling in B cells is important in disease progression. Recent studies have further highlighted that blocking TLR7 or MyD88 may be effective and therapeutic in human SLE (Brown et al., 2022). These results show that TLR7 plays an important role in the pathogenesis of SLE; hence, the role of TLR7 in B cells cannot be ignored.

Triptolide (TP) is the main active component isolated from *Tripterygium wilfordii* Hook F. *Tripterygium wilfordii* has excellent immunosuppressive and anti-inflammatory effects (Law et al., 2011; Ziaei and Halaby, 2016; Cheng et al., 2021; Cao et al., 2022), and it can be used for the treatment of SLE (Tao and Lipsky, 2000). TP has been reported to play a role in various diseases by inhibiting the NF-κB signaling pathway (Jiang et al., 2021; Liu et al., 2022). Moreover, it can inhibit the differentiation of B cells into CD138^+^CD27^+^ plasma cells and inhibit the secretion of IgA, IgG, and IgM by plasma cells (Zhao et al., 2018). In previous studies, triptolide has been shown to alleviate proteinuria and reduce serum anti-dsDNA antibody and TNF-α levels in MRL/lpr lupus mice (Huang et al., 2022). However, the specific mechanism of TP on SLE has not been fully investigated.

MicroRNAs (microRNA, miRNA) comprise a class of endogenous non-coding single-stranded RNA with a size of approximately 22 nucleotides, which mainly regulate gene expression at the post-transcriptional level. They can also participate in immune regulation (Dai and Ahmed, 2011). In addition, the activation of B cells and the expression of miRNAs may influence SLE. Studies have shown that TP can ameliorate lupus *via* the induction of miR-125a-5p-mediated Treg proliferation (Zhao et al., 2019). Therefore, studying the regulatory effect of TP on miRNA can further explore the therapeutic mechanism of TP on SLE.

Accordingly, this study aimed to investigate the therapeutic mechanism of TP on B cells in SLE based on the miR-146a/TLR7/NF-κB signaling pathway.

## Materials and methods

### Experimental animals

Fifteen SPF-grade female 8-week-old MRL/lpr mice were used in this study (production license number: SCXK (Shanghai) 2017-0005). MRL/Lpr mice, a well-established representative animal model of lupus pathogenesis, can produce lupus symptoms similar to human disease. They were randomly divided into the model (*n* = 5), low-dose TP (TP-L) (*n* = 5), and high-dose TP (TP-H) groups (*n* = 5). Based on the background of previous studies, the dose concentrations of TP administered were set at 30 μg/ml and 60 μg/ml. Mice in the TP-L group received 0.2 ml of a 30 μg/mL TP solution *via* gavage once a day. Mice in the TP-H group received 0.2 ml of a 60 μg/ml TP solution *via* gavage once a day. Mice in the model group received 0.2 ml of a normal saline solution *via* gavage once a day. Mice were dosed starting at the eighth week and continued for 8 weeks. The animal study was approved by the Laboratory Animal Management and Ethics Committee of the Zhejiang Chinese Medical University.

### Experimental cell and drugs

Raji cells, a B lymphocyte cell line, were cultured in RPMI 1640 medium supplemented with 10% fetal bovine serum. Triptolide was purchased from Shanghai Yuanye Biotechnology Co., Ltd. (product number: B20709-20mg). The has-miR-146a inhibitor and transfection reagent were purchased from Guangzhou Ribo Biotechnology Co., Ltd.

### Animal experiments and cell sorting

After 8 weeks of administration, the peripheral sera of mice were collected, and the levels of anti-dsDNA antibody, complement C3, complement C4, IgA, IgM, and IgG were detected using ELISA. Mouse Anti ds-DNA antibody elisa kit: MM-46291M1; Mouse Complement 3 elisa kit: MM-0354M1; Mouse Complement 4 elisa kit: MM-0343M1; Mouse Immunoglobulin A elisa kit: MM-0055M1; Mouse Immunoglobulin M elisa kit: MM-0058M1. Mouse Immunoglobulin G elisa kit: MM-0057M1. Purchased from MEIMIAN kit mall. CREA, BUN, ALT, AST, ALB, and ALP levels were detected using a fully automatic biochemical analyzer (HITACHI Automatic Analyzer 3100). After sacrificing *via* cervical dislocation, the kidneys of the mice were collected and fixed in 4% PFA for pathological examination. The spleens were also collected, immediately placed in RPMI 1640 medium, and ground, and the resulting cell solution was used for red blood cell lysis to obtain a single-cell suspension. Approximately 10^7^ cells were taken for flow antibody incubation, and the number of CD19^+^CD138^+^B220^−^ plasma cells was detected using a Beckman Cytoflex flowmeter. The remaining cells were sorted using CD43 (Ly-48) MicroBeads to obtain mouse spleen B cells. Reagent manufacturers and lot number: anti-mouse CD19: Biolegend, 115520; anti-mouse CD138: Biolegend, 142506; anti-mouse/human B220: Biolegend, 103247. CD43 (Ly-48) MicroBeads: Miltenyi, 130-049-801.

### Bioinformatic approach for predicting the targets and miRNAs of TP in treating SLE

“Triptolide” related targets were retrieved using the TCMSP database (https://old.tcmsp-e.com/tcmsp.php). The targets related to “systemic lupus erythematosus” were indexed using the GeneCards database (https://www.genecards.org/) and the Online Mendelian Genetics (OMIM) database (http://omim.org/). The targets of TP and SLE were intersected to obtain their overlapping targets. The overlapping targets were then analyzed in the STRING database, and the resulting PPI network was further visualized using Cytoscape 3.7.1 software. Gene Ontology (GO) and KEGG pathway enrichment analysis of overlapping targets were analyzed using R software (Yu et al., 2012). We used the R package clusterProfiler for analysis and defined *p* < 0.05.

The miRWalk and miRDB databases were used for the miRNA prediction of the overlapping targets. Briefly, the experimentally verified TP-related miRNAs were retrieved to determine their intersection, then the related miRNAs were obtained and visualized using Cytoscape 3.7.1 software.

### Detecting miR-146a expression using RT-qPCR

Total RNA from mouse spleen B cells was extracted, and reverse transcription-PCR (RT-PCR) was performed using a Bio-Rad Total RNA Kit. PCR amplification system: RNA Template 1 μg, miR-146a/U6 RT primer (5 μm) 1 μl, 5× Reverse Transcription Buffer 2 μl, RNase free H_2_O to make up to 10 μl. After mixing the above system, centrifuge briefly. The RT reaction program is as follows: 42°C for 60 min, 70°C for 10 min. The qPCR reaction system for miR-146a/ U6 comprised 10 μl 2×SYBR Green Mix, 2 μl RT product, 0.8 μl each of the forward and reverse PrimermiR-146a/U6 primers (primer concentration: 5 μM), and 6.4 μl dd H_2_O.

### Raji cell culture and transfection with a miR-146a inhibitor

In addition, after stable culture of Raji cells, TP solution was administered, and the expression of miR-146a and TLR7 mRNA was detected using qPCR at 3, 6, 12, and 24 h after drug intervention. Simultaneously, western blotting was used to detect the protein expressions of TLR7, MyD88, p-IRAK1, and p-NF-κBp65 following TP administration at the optimal intervention time. Moreover, after stable culture of Raji cells, Raji cells were transfected with 10, 20, 30, 50, 100 nM miR-146a inhibitor, respectively, and cultured at each concentration for 12, 24, 36, 48, and 72 h. The optimal inhibition time and concentration were determined using qPCR. TP solution was administered under the intervention of miR-146a inhibitor at the optimal concentration and time. The mRNA expressions of miR-146a and TLR7 were detected using qPCR, and the protein expressions of TLR7, MyD88, p-IRAK1, and p-NF-κBp65 were detected by western blot.

### Detecting the mRNA expression of related genes using RT-qPCR

Raji cells were collected after administration for total RNA extraction. Then, RT-qPCR was performed using a Bio-Rad Total RNA Kit. The sequences of the primers used were: TLR7, primer-F 5′-TCA​GCG​TCT​AAT​ATC​ACC​AGA​C-3′ and primer-R 5′-CAC​TGT​CTT​TTT​GCT​AAG​CTG​T-3′; IRAK1, primer-F 5′-GAG​AGT​GAC​GAG​AGC​CTA​GG-3′ and primer-R 5′-CTC​GAT​TCT​CCT​GCC​GTG​TC-3′; NF-κBp65, primer-F 5′-ACA​GAA​GCA​GGC​TGG​AGG​TAA​GG-3′ and primer-R 5′-GGA​CAA​TGC​CAG​TGC​CAT​ACA​GG-3′; and GAPDH, primer-F 5′-GAA​CGG​GAA​GCT​CAC​TGG-3′ and primer-R 5′-GCC​TGC​TTC​ACC​ACC​TTC​T-3′.

### Protein expression detection *via* western blot

Cells were lysed with RIPA lysis buffer to extract total protein. SDS-PAGE gels were prepared, followed by ECL chemiluminescence and exposure development. The primary antibodies (concentrations) used were anti-TLR7 (1:3000), anti-MyD88 (1:1000), anti-IRAK-1 (phospho-T209) (1:300), and phospho-NF-κBp65 antibodies (1:1000). Reagent manufacturers and lot number: PAGE Gel Rapid Preparation Kit: Yamei, PG112; anti-TLR7 antibody: abcam, ab124928; anti-MyD88 antibody: abcam, ab2064; anti-IRAK-1 (phospho-T209) antibody: abcam, ab218130; phospho-NF-κBp65 antibody (Ser536): CST, 3033S; Beta actin antibody: BIOKEN, BK7018.

### Statistical analysis

SPSS (Statistical Package for Social Sciences) 25.0 software, Flow Jo-V10 software, and GraphPad Prism 8 software were used to analyze and graph the experimental data. One-way ANOVA was used to compare multiple groups, and differences were considered statistically significant at *p* < 0.05.

## Results

### TP exerted therapeutic effects on MRL/lpr mice without obvious liver and kidney toxicity

**FIGURE 1:**
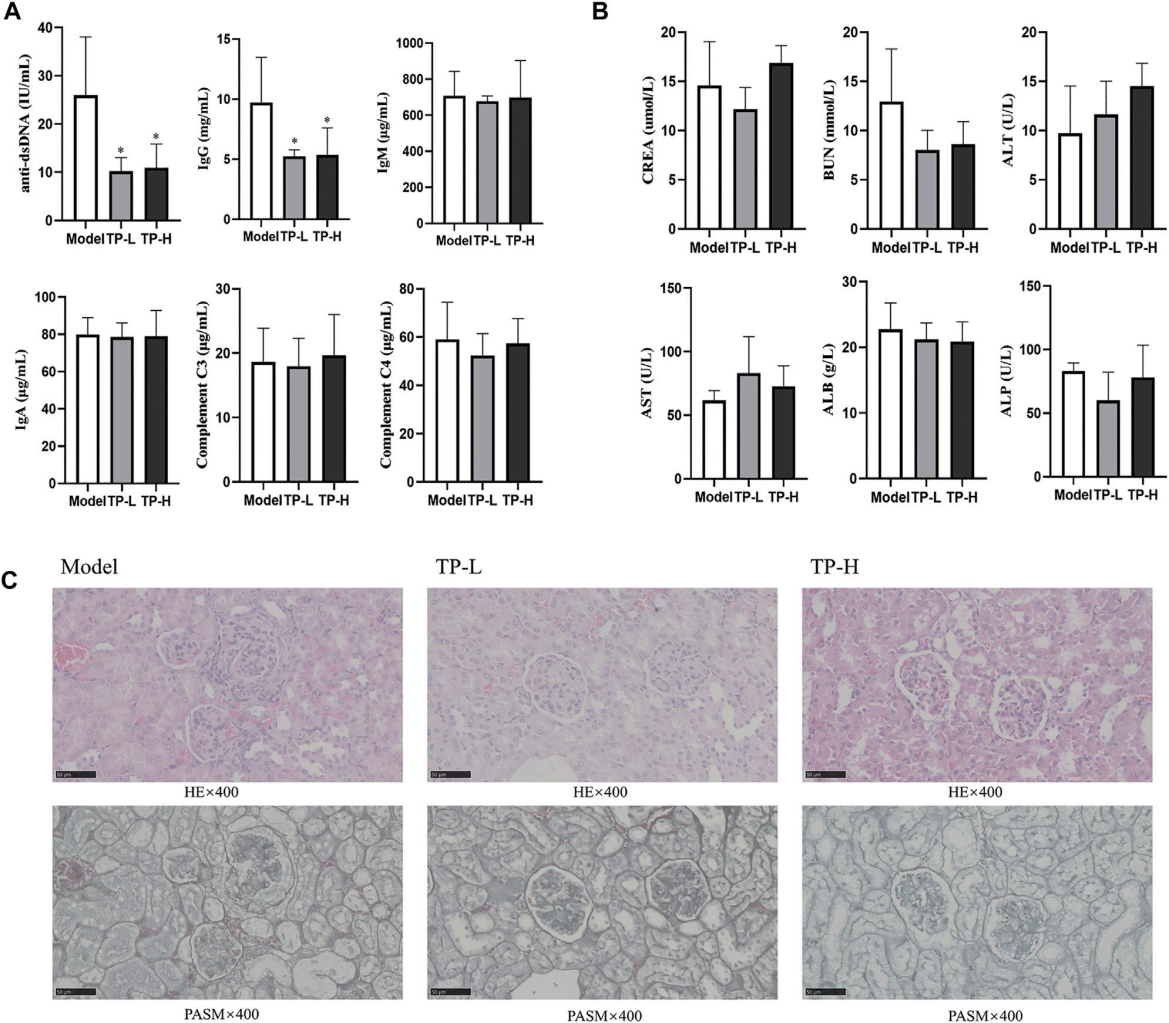
TP alleviates the disease condition in MRL/lpr mice. **(A)** Immune-related indexes in MRL/lpr mice. **(B)** Liver and kidney function indexes in MRL/lpr mice. **(C)** HE and PASM staining of the kidneys from MRL/lpr mice (Magnification ×400; Scale bar = 50 μm). (*n* = 5, *p < 0.05, **p < 0.01, ***p < 0.001).

**FIGURE 2:**
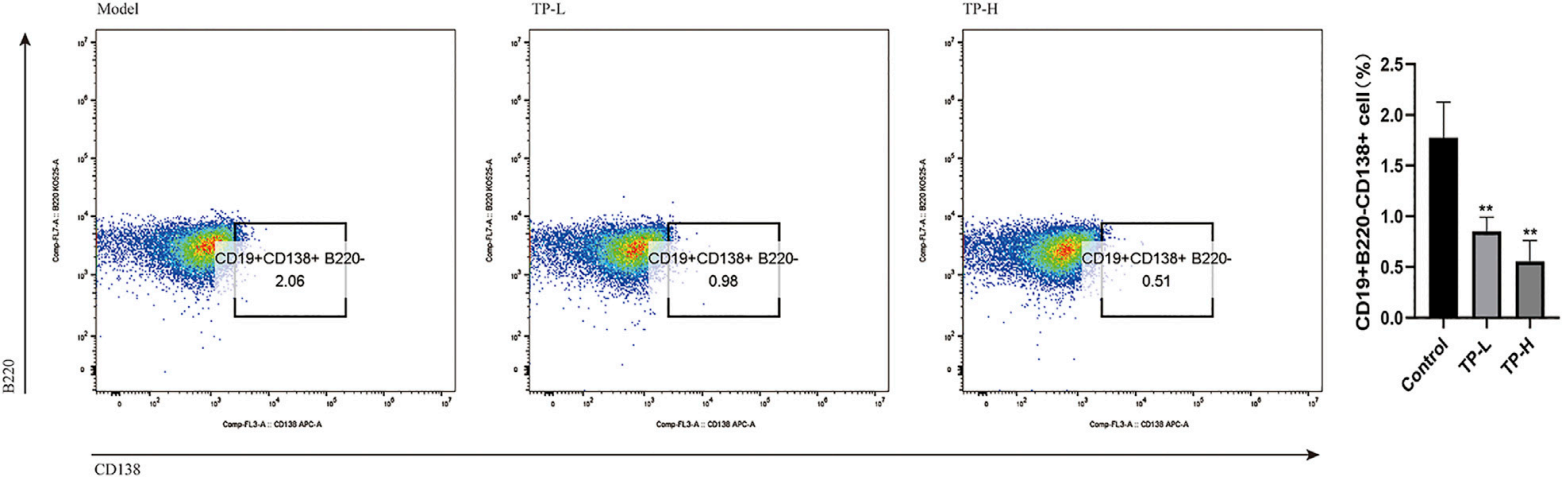
Spleen plasma cell ratio in MRL/lpr mice. Compared with the model group, the proportions of CD19^+^CD138^+^B220^−^ plasma cells in the TP-L and TP-H groups were significantly decreased (*p* < 0.01).

Compared with the model group, the levels of serum anti-dsDNA and IgG were decreased in the TP-L and TP-H groups (*p* < 0.05), while there were no significant differences in IgM, IgA, C3, and C4 levels (Figure 1A) and CREA, BUN, ALT, AST, ALB, and ALP levels between the model, TP-L, and TP-H groups (Figure 1B). Renal histopathological examination revealed inflammatory cell infiltration around the glomerulus and renal tubules, glomerular mesangial hyperplasia, capillary endothelial cell proliferation, and fibrocystic crescents in some glomeruli in the model group. In TP-L and TP-H mice, no fibrocystic crescents were found in the glomeruli, the infiltration of inflammatory cells around the glomerulus and renal tubules was significantly less than that of the model group, and the glomerular mesangial hyperplasia and proliferation of capillary endothelial cells were significantly better than that of the model group (Figure 1C). Compared with the model group, the proportions of CD19^+^CD138^+^B220^−^ plasma cells in the TP-L and TP-H groups were decreased (*p* < 0.01) (Figure 2).

### Bioinformatics prediction of the targets of TP in the treatment of SLE

**FIGURE 3:**
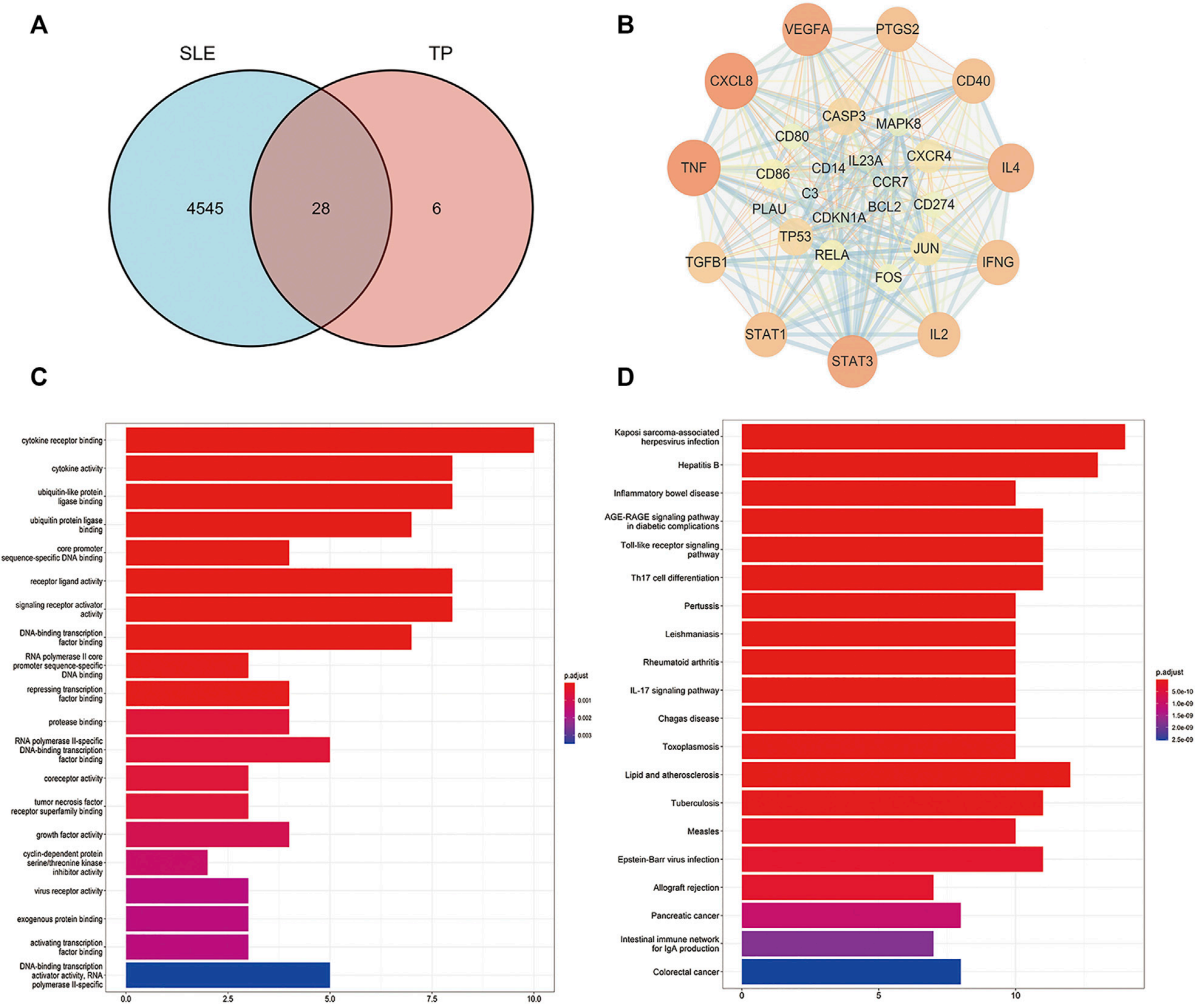
Network pharmacological analysis of the overlapping targets of TP and SLE. **(A)** Venn diagram of the compound targets of SLE and TP. **(B)** Visualization of the intersection targets of TP and SLE using Cytoscape 3.7.1 software. The dots represent the target of TP in the treatment of SLE and the edges represent the interactions between targets. **(C)** Gene ontology (GO) analysis of the overlapping targets of TP and SLE. **(D)** Kyoto Encyclopedia of Genes and Genomes (KEGG) analysis of the overlapping targets of TP and SLE.

Thirty-four TP-related targets were retrieved in the TCMSP database and 4,573 SLE-related targets were retrieved in the Genecards database. In total, 28 overlapping targets were obtained (Figure 3A), including TNF, C3, IFNG, TP53, TGFB1, IL4, IL2, VEGFA, CXCL8, STAT1, STAT3, CD40, CD274, CD80, CD86, PTGS2, CXCR4, IL23A, CCR7, BCL2, CDKN1A, CASP3, PLAU, FOS, REL, JUN, MAPK8, CD14. The interaction between the overlapping targets was analyzed in the STRING database, and a PPI network map was obtained. Cytoscape 3.7.1 software was used to visualize the network map (Figure 3B).

Next, we performed Gene Ontology (GO) analysis on the overlapping targets to further explore the mechanism of TP in the treatment of SLE. We found that the overlapping targets were mainly enriched in cytokine receptor binding, cytokine activity, ubiquitin-like protein ligase binding, ubiquitin protein ligase binding, core promoter sequence-specific DNA binding, receptor-ligand activity, signaling receptor activator activity, DNA-binding transcription factor binding, RNA polymerase II core promoter sequence-specific DNA binding, and repressing transcription factor binding (Figure 3C). We also performed a KEGG pathway enrichment analysis on the overlapping targets. We found that TP regulated SLE-related immune signaling pathways mainly involved in Toll-like receptor signaling, Th17 cell differentiation, and the IL-17 signaling pathway, among others (Figure 3D). Based on previous studies and our prediction of network pharmacological mechanisms, we selected the TLR-7 signaling pathway to verify the mechanism of TP in the treatment of SLE.

### Predicted miRNAs associated with the overlapping targets between TP and SLE

**FIGURE 4:**
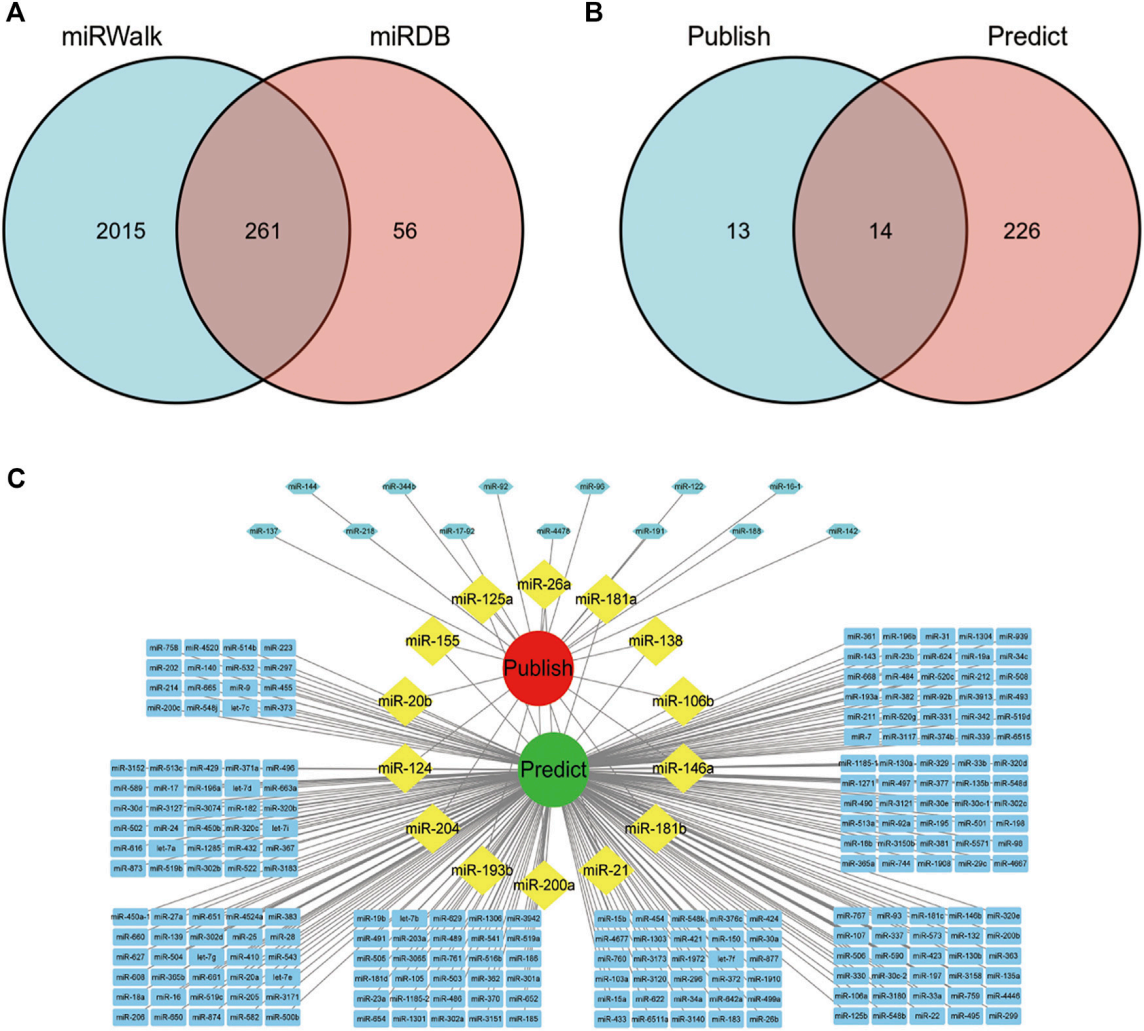
miRNAs acting on overlapping targets of SLE and TP. **(A)** Venn diagram of miRNAs predicted by the miRWalk and miRDB databases. **(B)** Venn diagram of TP-related miRNAs verified in literature and predicted in this paper. **(C)** Visualization of the network diagram of TP-related miRNAs verified in literature and predicted in this paper.

Next, we predicted related miRNAs acting on the overlapping targets of TP and SLE according to the miRWalk and miRDB databases. A total of 261 related miRNAs were obtained (Figure 4A). The predicted miRNAs were intersected with the TP-related miRNAs validated in the literature, and 14 miRNAs were obtained: miR-146a, miR-204, miR-181b, miR-20b, miR-125a, miR-181a, miR-200a, miR-124, miR-193b, miR-26a, miR-21, miR-138, miR-155, and miR-106b (Figure 4B). The results were visualized using Cytoscape 3.7.1 software (Figure 4C).

### TP upregulated miR-146a expression to inhibit the TLR7/NF-κB signaling pathway

**FIGURE 5:**
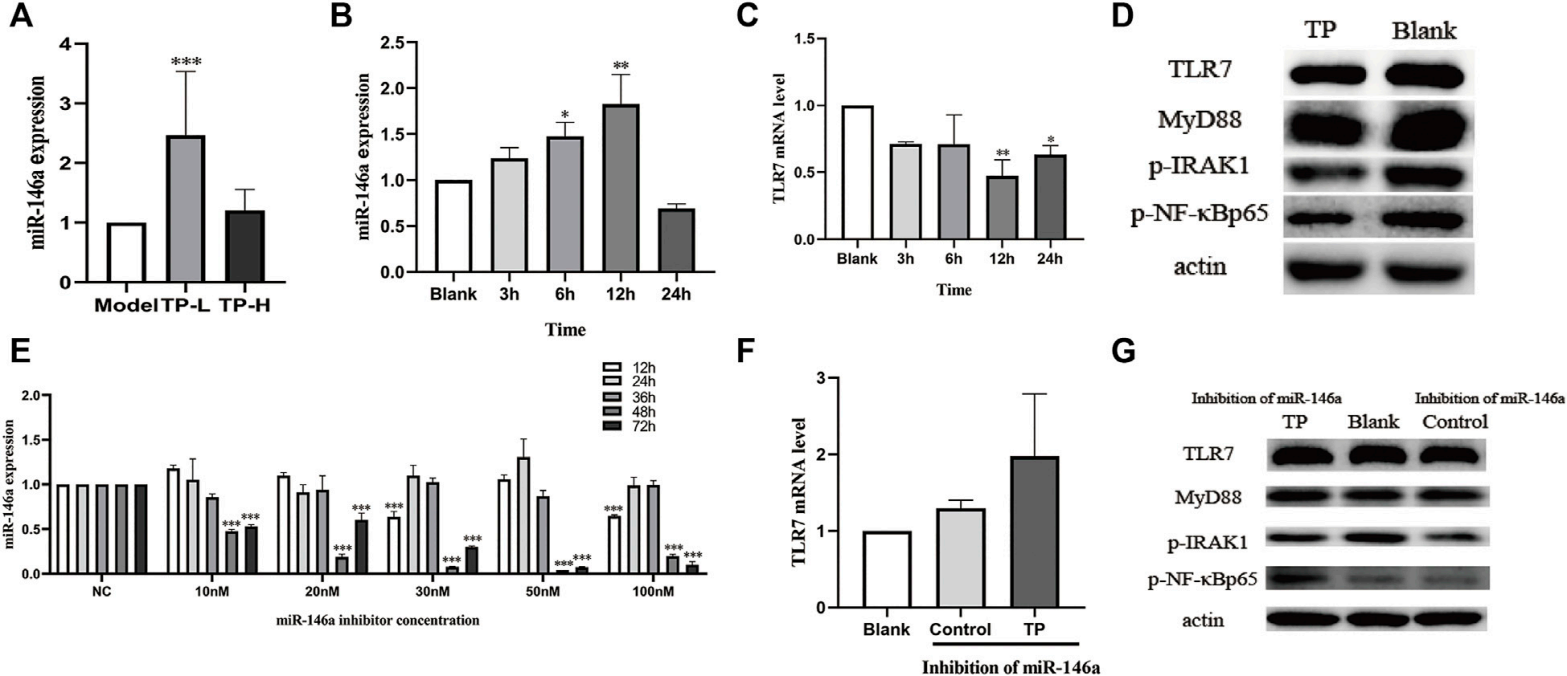
The effects of TP on miR-146a and the TLR7/NF-κB signaling pathway. **(A)** The effect of TP on miR-146a expression in the spleen of MRL/lpr mice. **(B)** The effects of TP on miR-146a expression in Raji cells at different time points. **(C)** The effect of TP on TLR7 mRNA expression in Raji cells at different time points. **(D)** The effect of TP on the protein expression of the TLR7/NF-κB signaling pathway in Raji cells. **(E)** Inhibitory efficiency of a miR-146a inhibitor on miR-146a in Raji cells at different concentrations and treatment durations. **(F)** The effect of TP on TLR7 mRNA expression in Raji cells upon treatment with a miR-146a inhibitor. **(G)** The effect of TP on the expression of TLR7 signaling pathway proteins in Raji cells treated with a miR-146a inhibitor. (*p < 0.05, **p < 0.01, ***p < 0.001).

Compared with the model group, the expression of miR-146a was upregulated in splenic B cells of MRL/lpr mice treated with TP-L and TP-H. The expression of miR-146a in the TP-L group was significantly increased (*p* < 0.05) (Figure 5A). Next, we carried out cell experiments using Raji cells and found that the expression of miR-146a was upregulated and the expression of TLR7 mRNA was downregulated after 12 h of TP administration (Figures 5B,C). Western blotting showed that TP could downregulate the protein expression levels of TLR7, MyD88, p-IRAK1, and p-NF-κBp65 (Figure 5D).

We then detected the inhibition efficiency of the miR-146a inhibitor in Raji cells and found that the optimal conditions were 50 nM concentration and 48 h of treatment (Figure 5E). Therefore, this condition was selected for subsequent experiments. In case of miR-146a inhibition in Raji cells, TLR7 mRNA was not downregulated in the TP groups (Figure 5F). Moreover, the protein expressions of TLR7, MyD88, p-IRAK1, and p-NF-κBp65 were also not downregulated (Figure 5G).

## Discussion

SLE is an autoimmune disease where B cell abnormalities are a key factor in its pathogenesis (Yap and Chan, 2019; Atisha-Fregoso et al., 2021). Plasma cells are the terminal differentiation link of B cells, which produce pathogenic antibodies in SLE (Malkiel et al., 2018). Toll-like receptors (TLRs) play pivotal roles in B cell activation and contribute to the pathogenesis of SLE (Jackson et al., 2014). For example, enhanced TLR7 signaling drives the aberrant survival of B cell receptor (BCR)-activated B cells (Brown et al., 2022). Moreover, TLR7 drives B cell response and the germinal center reaction involved in autoantibody production (Fillatreau et al., 2021). Notably, microRNAs also play a role in germinal center B cell production and their differentiation into antibody-secreting plasma cells (Meinzinger et al., 2018).

*Tripterygium wilfordii* Hook F has significant anti-inflammatory and immunosuppressive properties and is widely used in treating autoimmune and inflammatory diseases such as SLE (Song et al., 2020). TP is the main active ingredient in *Tripterygium wilfordii* Hook F. Previous studies have shown that TP can inhibit NF-κB transcription (Qiu and Kao, 2003), inhibit NF-κB/GADD45B signaling and attenuate proteinuria and podocyte apoptosis (Wang et al., 2018), and exert anti-inflammatory effects by inhibiting NF-κB activation (Bai et al., 2016). In this study, we found that an appropriate dose of TP could reduce the expression of ds-DNA and IgG, alleviate kidney damage, and reduce the proportion of CD19^+^CD138^+^B220^−^ plasma cells in MRL/lpr mice. Moreover, TP improved the disease condition of MRL/lpr mice without serious side effects. Accordingly, we further analyzed the mechanism of TP alleviating SLE.

We predicted *via* network pharmacology that the key pathways of the TP-and SLE-related overlapping targets include the Toll-like receptor signaling pathway. TLRs promote pathogen recognition by immune cells, producing pro-inflammatory cytokines and chemokines. TLRs are critical in the pathogenesis of SLE (Jackson et al., 2014). Specifically, the TLR7 in B cells plays an important role in regulating SLE, and regulating TLR7 expression can alleviate the symptoms of SLE (Fillatreau et al., 2021). TLRs are essential molecules that influence B cell activation signaling (Suthers and Sarantopoulos, 2017), as TLRs could decrease the activation threshold of B cells to some extent (Singh et al., 2016).

Accordingly, this study mainly focused on B cells and explored the effect of TP on the expression of TLR7 on B cells. We verified that TP could inhibit the expression of TLR7 mRNA and TLR7, MyD88, p-IRAK1, and p-NF-κB-p65 proteins in B cells. This evidence suggests that TP may inhibit the TLR7 signaling pathway in B cells and affect the differentiation of B cells into plasma cells.

miRNAs can regulate gene expression (Chen et al., 2013) and have potential roles in autoimmune regulation (O'Connell et al., 2010). The miRNA-mRNA network shows that hsa-miR-146a plays a vital role in pulmonary arterial hypertension secondary to SLE (Yao et al., 2021). Research has reported that the SLE risk variant rs2431697 likely causes SLE by disrupting a regulatory element and modulating miR-146a expression (Hou et al., 2021). The role of miR-146a in regulating the inflammatory response in SLE has been elucidated (Zhou et al., 2019), and studies have shown that miR-146a through the NF-κB signaling pathway reduces SLE-induced kidney injury in MRL/lpr mice (Fu et al., 2019). In this study, we predicted 14 miRNAs related to the overlapping targets of TP and SLE, including miR-146a. miR-146a is a major negative regulator of MyD88-dependent NF-κB activation and affects TLR7 expression (Chan et al., 2013). A decreased expression of miR-146a can downregulate the expression of IRAK1 and induce the development of SLE (Tang et al., 2009). Taganov and others confirmed that IRAK1 is the target of miR-146a and a critical downstream molecule in the TLR signaling pathway (Taganov et al., 2006). miR-146a can inhibit the expression of TLR7 and MyD88 (Karrich et al., 2013); hence, we further explored the effect of TP administration on TLR7 and miR-146a expression.

**FIGURE 6:**
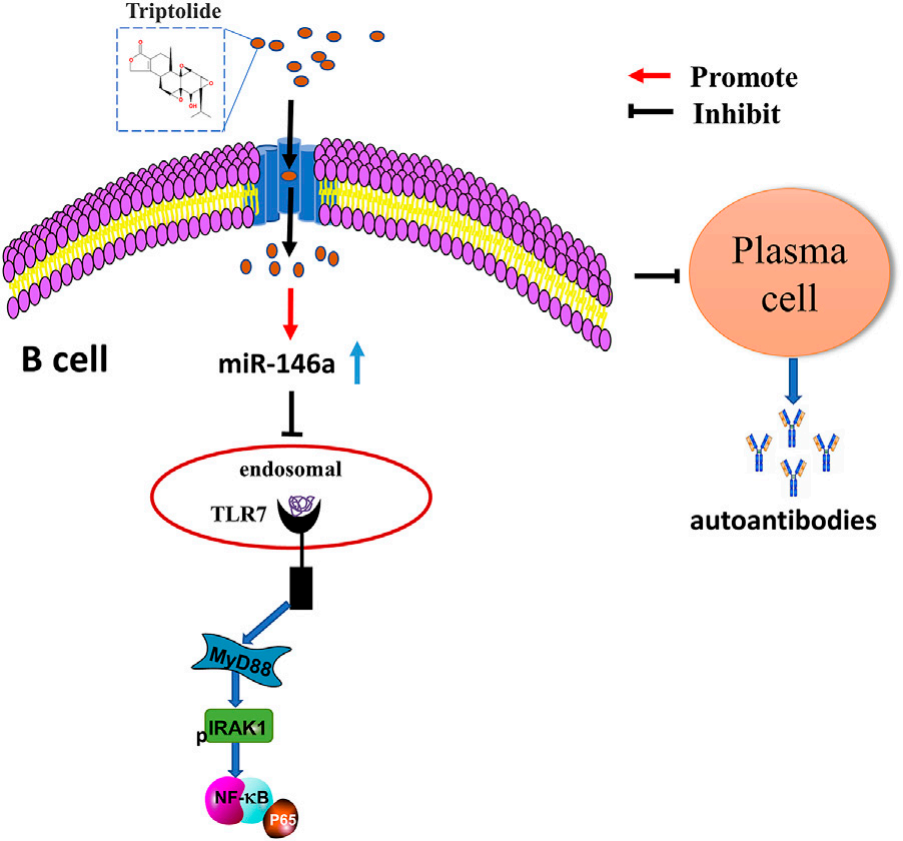
The underlying mechanism wherein TP affects the differentiation of B cells into plasma cells by regulating miR-146a to inhibit the TLR7/NF-κB signaling pathway.

We found that TP could upregulate miR-146a expression in the spleen B cells of MRL/lpr mice. Accordingly, we further explored the effect of TP on TLR7 and miR-146a. We found that TP no longer played a role in inhibiting the expression of TLR7 in B cells when the cells were treated with a miR-146a inhibitor. Thus, we concluded that TP could inhibit the TLR7-NF-κB signaling pathway by upregulating the expression of miR-146a in B cells (Figure 6).

Our results showed that TP alleviated SLE through the miR-146a-mediated downregulation of TLR7 expression. Herein, we propose a novel mechanism by which TP alleviates the disease conditions in MRL/lpr mice, which may provide support for the pharmacodynamic mechanisms underlying the application of TP in the treatment of SLE.

## Conclusion

In this paper, we confirmed that TP could alleviate SLE, and the mechanism of its efficacy may be elicited by affecting the differentiation of B cells into plasma cells by regulating miR-146a to inhibit the TLR7/NF-κB signaling pathway.

## Data Availability

The original contributions presented in the study are included in the article/Supplementary Material, further inquiries can be directed to the corresponding authors.
